# Preferences for treatment outcomes in rectal cancer: A discrete choice experiment among patients and healthy volunteers

**DOI:** 10.1111/codi.70021

**Published:** 2025-02-09

**Authors:** Anne Miles, Robert James Campbell Steele, Gemma Hutton, Stephen Morris

**Affiliations:** ^1^ School of Psychological Sciences Birkbeck University of London London UK; ^2^ School of Medicine University of Dundee Dundee UK; ^3^ Department of Public Health and Primary Care University of Cambridge Cambridge UK

**Keywords:** cancer, choice behaviour, low anterior resection syndrome, ostomy, patient preference, psycho‐oncology, rectal cancer, recurrence, risk, treatment outcome

## Abstract

**Aim:**

Treatment for rectal cancer can leave patients with a permanent stoma or bowel dysfunction. In this work we aimed to examine preferences for treatment outcomes among people with and without rectal cancer.

**Method:**

Our discrete choice experiment examined the effect of risk of cancer recurrence, presence of a stoma and bowel dysfunction on treatment preferences in 372 rectal cancer patients without a stoma, 269 with a stoma and 204 people without cancer.

**Results:**

Predictors of treatment preferences differed significantly between all groups (*p* < 0.0001). Avoiding a stoma was more important to stoma‐naïve groups, while avoiding bowel dysfunction was more important to those with superior function. Reducing the risk of recurrence was valued highly, and equally, across the groups.

**Conclusion:**

Experience of a stoma or bowel dysfunction resulted in higher tolerance of those treatment outcomes. Hearing from patients living with different treatment outcomes could help prepare newly diagnosed patients, and facilitate informed decision‐making where patients have a choice.

**What does this paper add to the literature?**

Treatment for rectal cancer can leave patients with a permanent stoma or bowel dysfunction. We examined preferences for treatment outcomes among people with and without rectal cancer. Experience of a stoma or bowel dysfunction resulted in higher tolerance of those treatment outcomes, while reducing the risk of recurrence was valued highly across all groups. Hearing from patients living with different treatment outcomes could help prepare newly diagnosed patients, and facilitate informed decision‐making where patients have a choice.

## INTRODUCTION

Colorectal cancer (CRC) is the third most common cancer in the world, and the second most common cause of cancer death, with rectal cancer making up a third of cases [1]. With incidence rates predicted to rise [2], more people will undergo treatment for CRC and must live with any treatment‐related side‐effects.

Most rectal cancer patients in developed countries receive sphincter‐sparing surgery such as low anterior resection (LAR) rather than abdominoperineal resection with permanent colostomy (APR) (e.g. [3, 4]). However, quality of life is not necessarily superior following LAR [5, 6, 7], mainly due to low anterior resection syndrome (LARS). LARS includes symptoms such as incontinence, frequency and urgency. Meta‐analyses have found that over 40% of patients experience major LARS one or more years postsurgery [8, 9]; this can have a significant impact on quality of life [10], particularly in social and emotional domains [11].

Although APR is often dictated by tumour location, some patients have a choice. However, patients are not always fully informed about the consequences of different options and do not always exercise their right to choose. Approximately two‐thirds of rectal cancer patients have reported leaving the choice to their surgeon [12]. People having LAR are often unaware of the potential for change in bowel function postsurgery [13], with 8% of patients resorting to a permanent stoma later, due to faecal incontinence [14].

While trade‐offs between faecal incontinence and presence of a stoma, as well as the amount of life expectancy people are willing to trade to avoid either a stoma or incontinence, have been examined before, the methods used (treatment trade‐off, time trade‐off [15] and threshold task [16]) do not allow for the simultaneous assessment of multiple attributes [17]. The latter can be achieved using a discrete choice experiment (DCE); however, a recent systematic review identified only one paper using a DCE to examine preferences in relation to surgery but focused on ‘attributes of trust’ between patients and surgeons, for example whether patients were being treated in a teaching or district hospital, rather than surgical outcomes [18]. The present study therefore used a DCE to examine preferences for rectal cancer treatment among people with no experience of rectal cancer and rectal cancer patients, both with and without a stoma, and examine the level of bowel dysfunction and risk of recurrence that the different groups would tolerate to avoid a stoma. We also aimed to examine whether preferences varied by age or gender.

## METHOD

This study received ethical approval from the Department of Psychological Sciences Research Ethics Committee of Birkbeck, University of London (no. 181997).

### Discrete choice experiment

DCEs present people with choices between two or more options that are described in terms of attributes. By systematically varying the levels of the attributes, the relative importance, and trade‐offs between them, can be determined. DCEs, which assess hypothetical choices, have been shown to produce reasonable predictions of choices in real life [19]. The main aim of this study was to examine what level of bowel function and risk of recurrence people would tolerate to avoid a permanent stoma. Risk of recurrence levels were varied from 1 to 16 in 100, based on 5‐year local recurrence rates following either APR or sphincter‐saving surgery [20, 21]. Risk of recurrence was presented as the number of people out of 100 who would experience a cancer recurrence if they had that treatment. Attributes and levels for bowel function were taken directly from the LARS scale, a validated, five‐item scale [22] (Table 1), considered the best measure of LARS [23] and the one used in meta‐analyses of LARS prevalence [8, 9]. Scores for the questionnaire can be summed and also categorized (no LARS, 0–20; minor LARS, 21–29; major LARS, 30–42). The attribute levels for bowel symptoms were examined individually as well as collectively by converting the levels into LARS scores using the LARS scoring instrument; for example, for the number of times the respondent would open their bowels, the attribute level ‘1–3 times per day’ was weighted 0 and the level ‘less than once per day’ was weighted 5.

**TABLE 1:** Attributes and attribute levels.

Attribute	Attribute levels			
Cannot control your flatus (wind)^a^	No, never	Yes, less than once per week	Yes, at least once per week	–
Accidental leakage of liquid stool^a^	No, never	Yes, less than once per week	Yes, at least once per week	–
Open your bowels^a^	Less than once per day (24 h)	1–3 times per day (24 h)	4–7 times per day (24 h)	More than 7 times per day (24 h)
Open your bowels again within 1 h of the last bowel opening^a^	No, never	Yes, less than once per week	Yes, at least once per week	–
Strong urge to open your bowels meaning you will have to rush to the toilet^a^	No, never	Yes, less than once per week	Yes, at least once per week	–
Chance cancer will come back	1 out of 100	6 out of 100	11 out of 100	16 out of 100
Stoma	No	Yes	–	–

^a^ Attributes and attribute levels taken from the low anterior resection syndrome (LARS) scale and used to compute the LARS score using the LARS scoring instructions.

The number of attributes and attribute levels result in a potential 2592 combinations (4^2^ × 3^4^ × 2^1^). With two options to choose from in each choice question, this gives a possible 6.7 million choices (2592 × 2591). To reduce the number of choice sets presented to an individual, an orthogonal fractional design was used to generate treatment A. Treatment B was generated using a rotational design, whereby the level for each attribute was moved up one (e.g. if risk of recurrence was 6 out of 100 for treatment A, it was 11 out of 100 for treatment B). The orthogonal plan was used to produce 32 choice‐sets. To reduce the number of choice‐sets that each participant was required to consider we divided these into four groups of eight choice‐sets. Participants were randomly assigned to one of these and the order of presentation of choices within each block was also randomized.

We aimed to obtain a minimum of 200 participants per group because sample sizes below 150 show a rapid decrease in the precision of estimates, while samples sizes above 300 offer relatively little additional precision [24].

Feedback on the DCE and the questionnaire was obtained from five people who had had rectal cancer, recruited from cancer charity websites, and minor changes were made to the explanation of the attributes.

### Measures

All data were self‐reported. Age, gender, highest educational qualification, ethnicity, employment status and self‐rated health were measured for all participants. Self‐rated health was assessed with the question and response options used in the England and Wales Census 2021 [25]. Self‐rated health is strongly associated with having a history of chronic conditions as identified in electronic health records. For example, 88.2% of people who rated their health as ‘very bad’ had a history of at least one of 15 chronic health conditions, compared with 26.1% of those who rated their health as ‘very good’ [25].

Rectal cancer patients were asked about the presence of a stoma, using the question on the FACT‐C version 4 ‘Do you have an ostomy appliance?’ (yes/no) [26]. Comorbidities were measured using the Self‐Administered Comorbidities Questionnaire [27]. This measure lists 13 conditions (with an optional section where people can add other medical problems). For each condition, people are asked to indicate whether they have the problem, and, if yes, to answer whether they receive treatment for it and whether it limits their activities. Response options are yes/no, with a point awarded to each ‘yes’ response. An individual can receive a maximum of 3 points per medical condition. Only the closed items were used, and cancer was removed from the list, meaning people could score a maximum of 36 points. Time since diagnosis was calculated in years, with people asked to indicate the year their cancer was diagnosed, which was subtracted from the year they were invited to complete the study. Personal bowel function in rectal cancer patients without a stoma and participants without cancer was assessed using the LARS scale [21] (Table 2).

**TABLE 2:** Demographic and health scores by group (means (SD) or valid per cent (*n*)).

	Overall sample (*n* = 873)	Cancer no stoma (*n* = 372)	Cancer with stoma (*n* = 269)	No cancer (*n* = 204)
Age (years)^a^, ^b^	65.4 (SD 9.5)	66.3 (SD 9.5)	67.7 (SD 9.6)	60.7 (SD 7.6)
Gender (% male)^a^, ^b^	66.7 (562)	68.5	70.9	58.1
Educational level^a^, ^b^				
Degree level or equivalent	44.9 (377)	47.0 (174)	49.8 (133)	34.5 (70)
Below degree level	45.5 (382)	44.3 (164)	37.1 (99)	58.6 (119)
None	5.8 (49)	5.9 (22)	6.0 (16)	5.4 (11)
Prefer not to say	3.8 (32)	2.7 (10)	7.1 (17)	1.5 (3)
Ethnicity (% white)^a^, ^b^	96.9 (816)	97.8	98.5	93.1
Employment^a^, ^b^, ^c^, ^d^				
Employed	28.1 (237)	25.3 (94)	19.4 (52)	44.8 (91)
Self‐employed	6.4 (54)	8.6 (32)	4.9 (13)	4.4 (9)
Unemployed	2.6 (22)	0.8 (3)	3.0 (8)	5.4 (11)
Disabled/too ill to work	3.7 (31)	1.1 (4)	4.5 (12)	7.4 (15)
Retired	56.9 (479)	62.0 (230)	67.5 (181)	33.5 (68)
Home‐maker/student	1.9 (16)	1.6 (6)	0.4 (1)	4.4 (9)
Prefer not to say	0.4 (3)	0.5 (2)	0.4 (1)	0 (0)
Self‐rated health^a^, ^b^, ^e^				
Very bad	0.6 (5)	0.3 (1)	0.7 (2)	1.0 (2)
Bad	2.9 (25)	1.6 (6)	4.1 (11)	3.9 (8)
Fair	25.0 (212)	20.4 (76)	21.9 (59)	37.3 (76)
Good	45.8 (388)	43.3 (161)	47.6 (128)	48.5 (99)
Very good	25.7 (218)	34.4 (128)	25.7 (69)	9.3 (19)
Comorbidities	–	2.3 (2.6)	2.6 (2.6)	‐
Time since diagnosis (years)	–	1.9 (SD 1.8)	2.1 (SD 1.9)	‐
Personal LARS score^b^	–	26.6 (11.0) (median = 29.0)	‐	15.1 (11.1) (median = 14.0)
Proportion with major LARS^b^	–	47.7 (177)	‐	11.8 (24)

Abbreviation: LARS, low anterior resection syndrome.

^a^ Significant difference between cancer with stoma and no cancer.

^b^ Significant difference between cancer no stoma and no cancer.

^c^ Grouped into three: employed (full‐time, part‐time or self‐employed), retired or other (unemployed, student, disabled or too ill to work, fulltime homemaker).

^d^ Significant difference between cancer no stoma and cancer with stoma.

^e^ Grouped into good/very good versus fair/bad/very bad.

### Participants

Patient recruitment was carried out by Picker Institute Europe which runs the National Cancer Patient Experience Survey (NCPES). The NCPES is usually sent every year to all patients in England aged 16 or over with a primary diagnosis of cancer who attended hospital for cancer‐related treatment as either an inpatient or a day case patient and who were discharged between April and June of that year. Inclusion criteria for our study were patients with an ICD‐10 code of C20 (rectal cancer diagnosis) who had consented to be contacted again. Exclusion criteria were respondents with metastatic disease or those who were identified as deceased following a DBS check. The first recruitment wave took place in 2021 (respondents to the 2019 NCPES) and the second in 2022 (respondents to the 2021 NCPES). A sample of older adults who had not had any form of cancer were recruited via an online company, who offer people rewards in exchange for survey completion. Participants gave informed consent.

### Statistical analysis

**FIGURE 1:**
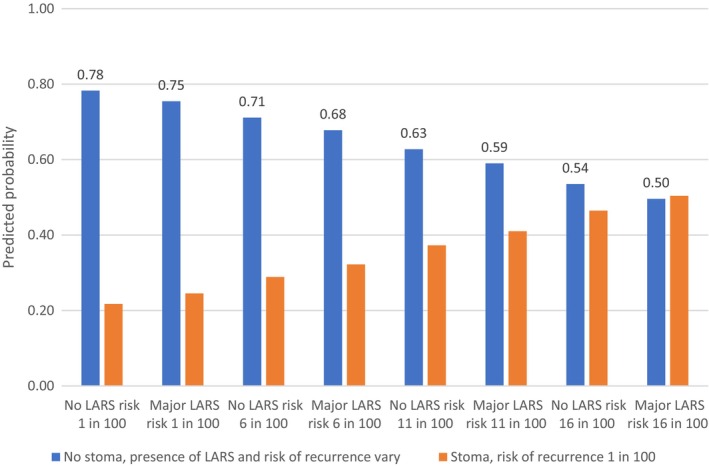
Predicted probability of choosing no stoma with varying levels of (LARS) and risk of recurrence, compared with choosing a stoma with a 1% risk of recurrence for the cancer no stoma group.

**FIGURE 2:**
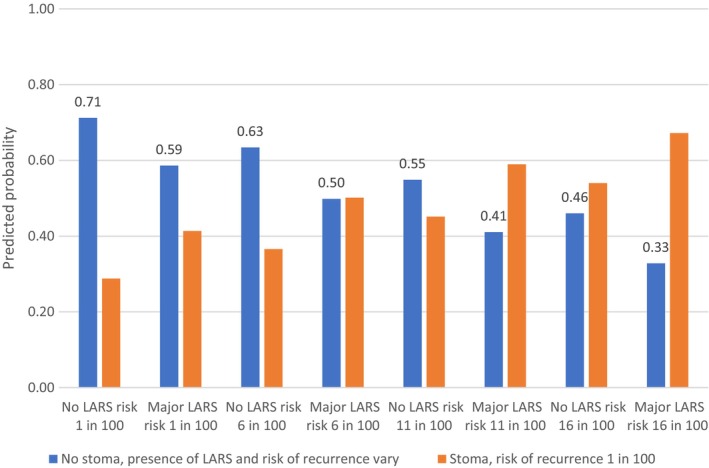
Predicted probability of choosing no stoma with varying levels of low anterior resection syndrome (LARS) and risk of recurrence, compared with choosing a stoma with a 1% risk of recurrence for the no cancer group.

**FIGURE 3:**
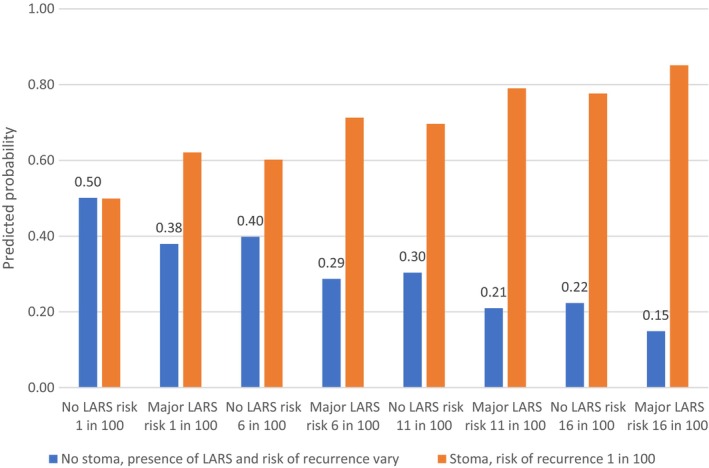
Predicted probability of choosing no stoma with varying levels of low anterior resection syndrome (LARS) and risk of recurrence, compared with choosing a stoma with a 1% risk of recurrence for the cancer with stoma group.

The data were analysed using a conditional logit regression model (fixed‐effects logit) in Stata version 16. The outcome variable was treatment choice (A or B) and the predictors were the attributes. Regression coefficients were used to calculate marginal rates of substitution (MRS). MRS allow direct assessment of how much of one attribute (the denominator) participants are willing to trade for one unit of another attribute (the numerator), enabling comparison of different attributes on a common scale. MRS were computed using both risk of cancer recurrence and the LARS score. The relative importance of attributes was computed for continuous variables by subtracting the product of the beta coefficient for the lowest level from that for the highest level. For categorical variables, this was calculated as the difference in the coefficients between the best or most preferred level of each attribute and the worst or least preferred level of the same attribute [28]. We used the regression coefficients in Table S1 (see Data S1) to calculate the predicted probabilities of choosing a permanent stoma, assuming a risk of recurrence of 1%, compared with no stoma at varying levels of risk of recurrence and presence of major LARS (Figures 1, 2, 3). We also varied the risk of recurrence until the probability of choosing a permanent stoma (yes/no) was equivalent (i.e. 50%).

Subgroup analyses were performed by cancer and stoma status, age (using a median split) and gender. Exploratory analysis was conducted on presence of LARS (among the cancer no stoma group only). Only participants who completed the DCE were included in the study. Twenty‐eight of the rectal cancer patients did not answer the question about having an ostomy appliance so were excluded from the subgroup analysis. For all other variables, missing data were <5%. Differences between the groups on demographic and health variables were assessed using chi‐square for frequency data and independent *t*‐tests for continuous data.

## RESULTS

A total of 1138 rectal cancer patients were invited to take part in the first recruitment wave in 2021, and 949 were invited in the second recruitment wave in 2022. Only patients who had completed the DCE were included in this study (*n* = 669, a response rate of 32.1%). In addition, 204 people without cancer also completed the DCE. Participants without cancer were significantly younger, more likely to be female and reported poorer self‐rated health compared with the two cancer groups, with no difference in age, gender or self‐rated health between the latter. The cancer no stoma group reported worse bowel function than the no cancer group (see Table 2 for all group differences).

The importance of treatment attributes differed significantly between all three groups: cancer patients without a stoma (CNS) versus patients with a stoma (CS) *χ* ^2^ (13) = 393.60 (*p* < 0.0001); CNS versus people without cancer (NC) *χ* ^2^ (13) = 59.86 (*p* < 0.0001); and CS versus NC *χ* ^2^ (13) = 159.91 (*p* < 0.0001). There were also significant differences by age (*χ* ^2^ (13) = 64.19; *p* < 0.0001) but not gender (*χ* ^2^ (13) = 14.66; *p* = 0.3290). The importance of treatment attributes in the CNS group did not significantly differ by LARS status (no LARS versus major LARS: *χ* ^2^ (13) = 20.16; *p* = 0.0912). All attributes significantly predicted treatment preferences except for leakage of wind (CNS, NC, younger participants), incomplete emptying (CNS, CS, older participants) and presence of a stoma (CS group only) (Tables 3 and S2).

**TABLE 3:** Results of conditional logit regression analysis by group, for all attributes (coefficients and 95% CIs) and marginal rates of substitution.

Attribute and attribute levels	All participants	Cancer no stoma	Cancer with stoma	No cancer
Leakage of wind				
No, never	–	–	–	–
Yes, less than once per week	−0.090 (−0.161, −0.020)^a^	−0.0341 (−0.152 to 0.083)	−0.181 (−0.307 to −0.055)^a^	−0.092 (−0.245 to 0.061)
Yes, at least once per week	−0.136 (−0.217 to −0.056)^a^	−0.0432 (−0.180 to 0.094)	−0.241 (−0.385 to −0.097)^a^	−0.154 (−0.326 to 0.0182)
Leakage of liquid stool				
No, never	–	–	–	–
Yes, less than once per week	−0.332 (−0.402 to −0.262)^a^	−0.264 (−0.383 to −0.146)^a^	−0.361 (−0.484 to −0.237)^a^	−0.334 (−0.486 to −0.181)^a^
Yes, at least once per week	−0.434 (−0.518 to −0.349)^a^, ^b^	−0.328 (−0.472 to −0.183)^a^	−0.498 (−0.648 to −0.348)^a^	−0.599 (−0.783 to −0.416)^a^, ^b^, ^d^
Need to empty bowels again within 1 h of emptying (incomplete emptying)				
No, never	–	–	–	–
Yes, less than once per week	−0.098 (−0.168 to −0.029)^a^	0.007 (−0.113 to 0.127)	−0.117 (−0.240 to 0.006)	−0.289 (−0.442 to −0.136)^a^, ^d^
Yes, at least once per week	−0.076 (−0.159 to 0.008)	−0.099 (−0.240 to 0.041)	0.022 (−0.128 to 0.172)	−0.204^a^ (−0.385 to −0.024)
Need to rush to the toilet				
No, never	–	–	–	–
Yes, less than once per week	−0.227 (−0.296 to −0.158)^a^	−0.133 (−0.250 to −0.017)^a^	−0.346 (−0.467 to −0.225)^a^, ^d^	−0.285 (−0.437 to −0.133)^a^
Yes, at least once per week	−0.217 (−0.301 to −0.134)^a^	−0.0542 (−0.195 to 0.0874)	−0.347 (−0.495 to −0.200)^a^, ^d^	−0.364 (−0.545 to −0.184)^a^, ^d^
Number of times open bowel				
1–3 times per day	–	–	–	–
Less than once per day	−0.066 (−0.164 to 0.031)	−0.182 (−0.349 to −0.014)^a^	0.085 (−0.079 to 0.250)^d^	−0.064 (−0.279 to 0.151)
4–7 times per day	−0.345 (−0.437 to −0.254)^a^	−0.300 (−0.456 to −0.144)^a^	−0.312 (−0.475 to −0.148)^a^	−0.351 (−0.550 to −0.152)^a^
More than 7 times per day	−0.585 (−0.694 to −0.476)^a^, ^c^	−0.507 (−0.694 to −0.320)^a^, ^c^	−0.503 (−0.694 to −0.312)^a^, ^c^	−0.739 (−0.978 to −0.501)^a^, ^c^
Risk of recurrence	−7.402 (−8.051 to −6.753)^a^	−7.884 (−9.041 to −6.726)^a^	−8.112 (−9.299 to −6.926)^a^	−7.107 (−8.489 to −5.725)^a^
Presence of a stoma				
No	–	–	–	–
Yes	−0.779 (−0.834 to −0.723)^a^	−1.290^a^ (−1.385 to −1.195)	−0.034 (−0.130 to 0.062)^d^	−0.983 (−1.107 to −0.859)^a^, ^b^, ^e^
Observations/respondents	13 968/873	5952/372	4304/269	3264/204
Marginal rates of substitution				
Increase in risk of recurrence to avoid a stoma	10.5%	16.3%	–	13.8%
Increase in LARS score to avoid a stoma	40.8	125.7	–	32.8
Increase in risk of recurrence to avoid minor LARS	4.0%	3.1%	4.1%	7.1%
Increase in risk of recurrence to avoid major LARS	4.6%	2.1%	6%	7.8%

*Note*: – indicates stoma was not a significant predictor so the MRS was not computed.

Abbreviation: LARS, Low anterior resection syndrome.

^a^ Significantly different from reference category [the value in brackets].

^b^ Significantly different from the level “yes, less than once a week.”

^c^ Significantly different from the level “4 to 7 times per day.”

^d^ Significantly different from the equivalent level in the cancer no stoma group.

^e^ Significant difference between cancer with stoma and no cancer groups.

### Cancer no stoma versus cancer stoma

The CNS group were more averse to a permanent stoma and the presence of constipation than the CS group. In the CS group, these two variables did not significantly influence treatment preferences. In contrast, the CS group were more averse to bowel urgency than the CNS group.

### Cancer no stoma versus no cancer

The CNS group were also more averse to a permanent stoma than the NC group. However, the NC group were more averse to poor bowel function (incontinence for liquid stool, incomplete emptying and urgency) than the CNS group.

### Cancer stoma versus no cancer

The NC group were more averse to a permanent stoma than the CS group.

### Older versus younger age

Risk of recurrence, presence of a stoma, incontinence for liquid stool, urgency and incomplete emptying had a stronger influence on the preferences of younger compared with older participants.

### Bowel function severity

Significant differences between more versus less severe symptoms were only observed for frequency (across all groups), and incontinence for liquid stool (only in the younger participants and the NC group) (Table S3). Consistent with this, there was no evidence of a difference between minor versus major LARS within any of the groups. However, between groups, the presence of major LARS was a stronger predictor of treatment preference in the CS group compared with the CNS group, and both minor and major LARS were more important predictors of treatment preference in the NC than the CNS group, and in younger compared with older age groups (Table S1).

### Marginal rates of substitution

The CNS group were only willing to accept a stoma if it conferred a 16.3% reduction in the risk of recurrence or a reduction in LARS symptoms of 125.7 (in other words they would accept a maximum LARS score). Similar, although lower, figures were observed in the NC group, particularly for LARS symptoms (13.8% and 32.8%). Presence of a stoma did not significantly predict treatment preferences in the CS group, so MRS were not computed (Table 3 and Table S4 for coefficients).

The NC group were more averse to both minor and major LARS than the other two groups, for example they only accepted major LARS if there was a 7.8% reduction in risk of recurrence compared with a 6.0% reduction in the CS group and 2.1% in the CNS group. Younger participants were willing to trade a higher risk of recurrence to avoid a stoma than older people (11.4% vs. 9.6%) and to avoid both minor (5.2% vs. 2.9%) and major LARS (5.6% vs. 3.7%) (see Table S1 for coefficients).

### Relative importance

Risk of recurrence was the most important attribute for both the CS and NC groups, and younger and older participants. It was the second most important attribute for the CNS group. Presence of a stoma was the most important attribute for the CNS group, and the second most important attribute for the other groups except the CS group, where it was least important. The relative ranking of the following bowel symptoms was the same across the three groups, with frequency the most important, followed by incontinence for liquid stool and then the need to rush to the toilet (see Table S5).

### Predicted probabilities

The CNS group were more likely to choose no stoma even if this meant a higher risk of cancer recurrence and the presence of major LARS. For example, 59% chose no stoma with major LARS and a risk of recurrence of 11% rather than a stoma and a risk of recurrence of 1% (see Figure 1). The two options of stoma versus no stoma (assuming no LARS) were only viewed as equivalent if having a stoma conferred a 17% lower risk of recurrence. In the presence of major LARS, they were only viewed as equivalent if having a stoma conferred a 15% lower risk of recurrence.

The NC group were also more likely to choose no stoma even if this meant a higher risk of cancer recurrence or major LARS. For example, 63% chose no stoma with a risk of recurrence of 6% assuming no LARS, and 59% chose no stoma with major LARS with a risk of recurrence of 1%. However, if no stoma meant both a higher risk of recurrence and the presence of major LARS, a permanent stoma became equally or more desirable. For example, at 6% risk of recurrence and the presence of major LARS, no stoma was chosen at the same rate as a stoma with 1% risk of recurrence (see Figure 2). The NC group were therefore less averse to a stoma and more averse to bowel problems than the CNS group. The two options of stoma versus no stoma were only viewed as equivalent if having a stoma meant a much lower risk of recurrence of 12.5%, assuming no LARS in the no stoma condition. Where no stoma also came with major LARS, the rates of acceptance were only the same if having a stoma meant a 5% lower risk of recurrence.

There was a higher tolerance for a stoma in the CS group, who selected a stoma at the same rate as no stoma if both had an equal probability of cancer recurrence (1%) and no stoma also came with no LARS. In the event of major LARS, a permanent stoma was preferred, with 62% choosing this option (see Figure 3). No stoma with major LARS was only selected at the same rate as a stoma if the former resulted in a 6% lower risk of recurrence.

Younger people were slightly more averse to having a permanent stoma than older people, with 72% of younger people choosing no stoma over a stoma compared with 64% of older people (assuming a 1% risk of recurrence and no LARS). Younger people accepted a permanent stoma at the same rate as no stoma if having a stoma conferred an 11% lower risk of recurrence compared with 9% in older people (assuming no LARS) (see Figures S1 and S2).

## DISCUSSION

Predictors of treatment preferences differed significantly between all three groups. While avoidance of a stoma was the first or second most important attribute in the no stoma groups (CNS and NC, respectively) it was the least important attribute in the CS group. Reducing the risk of recurrence was either the first (CS and NC groups) or second (CNS group) most important attribute and was valued equally across the groups. Our results showed that people were willing to accept an increase in risk of cancer recurrence to avoid treatment outcomes such as a stoma or poor bowel function, but that this varied by experience. Participants without a stoma were much more averse to having one: the CNS group accepted either an increased risk of cancer recurrence of 16.3% or major LARS to avoid a permanent stoma. Similar, although slightly lower, scores were observed in the NC group. The magnitude of the trade‐offs between risk of recurrence and a stoma we observed among no stoma participants are slightly higher than those reported by Lee et al. [16], who found noncancer patients accepted a 10% increased risk of margin involvement to avoid a stoma [16].

In contrast, in the CS group, the presence of a permanent stoma did not significantly influence treatment preferences when risk of cancer recurrence and bowel symptoms were controlled for. The predicted probabilities showed that the CS group were as likely to choose a permanent stoma as they were to choose no stoma, even when risk of recurrence was equivalent, assuming no significant bowel dysfunction (no LARS). In the presence of major LARS, a permanent stoma was preferred. This is consistent with recent research showing high levels of stoma acceptance 1–2 years postsurgery, with 83% of patients reporting they were not bothered by their stoma 2 years after surgery [29].

There was also higher tolerance of LARS among people more likely to have experience of it: the NC group only accepted major LARS for a 7.8% reduction in risk of recurrence compared with a 2.1% reduction in risk of recurrence in the CNS group, approximately half of whom had major LARS.

Higher tolerance for either stoma or bowel dysfunction among people with experience of these outcomes is consistent with previous research [12, 15]. For example, Bossema et al. [15] found the median percentage of life expectancy people that were willing to give up to be in good health instead of having a permanent stoma was 8% for APR patients and 37% for LAR patients, while 32% of LAR patients were willing to accept certain daily faecal incontinence in order to avoid a permanent stoma, compared with 2% of APR patients.

The relative ranking of the top three bowel symptoms was the same across the groups, with frequency (diarrhoea) the most important variable, followed by incontinence for liquid stool and the need to rush to the toilet. Treatment preferences favoured no LARS, but within the groups there were no significant differences between minor versus major LARS. This is consistent with our findings that the level of severity of bowel dysfunction was only important for two of the five bowel symptoms, one of which (incontinence for liquid stool) receives the same score in the LARS scoring system regardless of whether it occurs less than once a week or more than once a week.

In contrast to the findings of Lee et al. [16] we found that preferences for treatment outcomes also varied by age, with younger participants influenced more strongly by most of the attributes. However, in our sample, the NC group were significantly younger than the two cancer groups, meaning younger age and absence of cancer were associated, and the separate influence of the two variables could not be fully determined.

### Limitations

Strengths of our study include a large sample size, several subgroups to enable comparison between groups with experience of different treatment outcomes, and the exploration of seven treatment attributes. However, the following limitations should be noted. Our data on stoma status were self‐reported and we could not examine the effect of surgical procedure (APR or Hartmann's procedure where a rectal stump remains in place), stoma duration (permanent versus temporary stoma) or stoma type (ileostomy or a colostomy) on preferences. Both type of surgery and type of stoma influence complication rates [30, 31]. Hartmann's procedure has been shown to increase the risk of intra‐abdominal infection, while APR has been shown to increase the risk of overall complications [30]. If our sample contains a high rate of patients in receipt of Hartmann's procedure, this could have led to higher stoma acceptance among patients with a stoma in our study. It is also possible that some of the CNS group had had a stoma reversal and such prior experience might have led to greater aversion to a permanent stoma in this group. Adaptation to a stoma takes time [32] and may not occur with a temporary stoma. In addition, the experience of certain complications may be particularly aversive. While some complications are more frequent in patients receiving a colostomy (such as prolapse), others, such as dehydration, appear more frequent in those receiving an ileostomy [31]. Dehydration is the most common cause of readmission to hospital following an ileostomy [33] and future research could examine the effect of stoma type and complications on patient preferences.

Our DCE did not include outcomes such sexual dysfunction or surgical complications, which can adversely impact quality of life (e.g. Kreisel et al. [34]). However, adding more attributes would have made the DCE overly complex, and reducing the number of bowel function attributes would have prevented us from being able to generate LARS scores. Nevertheless, future research could focus on a smaller number of bowel functions, such as frequency and incontinence for liquid stool, as these emerged as the two most important bowel function attributes in our study, and would enable fuller consideration of other treatment outcomes.

The importance of treatment attributes in the CNS group showed a nonsignificant trend by LARS status (no LARS versus major LARS) and a higher sample size in this group may have found significant differences. All participants were based in England, and we only received responses from a third of eligible participants, the majority of whom had educational qualifications and were of white ethnicity. All these factors may have increased stoma acceptance in the CS group. Stoma acceptance is higher among more educated groups [35] and quality of life following a permanent stoma is better among northern Europeans compared with southern Europeans and people of Arabic origin [36]. While stoma creation is more frequent in low‐ and middle‐income countries than in high‐income countries [37] access to stoma counselling and affordable stoma care supplies is poor and such factors improve patient acceptance of a stoma [38].

The NC group were younger, more likely to be female and reported poorer health than the two cancer groups. Gender did not influence treatment preferences in our study, and previous research on the importance of treatment outcomes in colorectal patients also found no differences by gender in the perceived importance of attaining a complete cure, avoiding a stoma or having ‘reliable control of defecation’ [35] so this difference is unlikely to have influenced the findings in the NC group. In terms of age, we observed a different pattern of preferences by age versus cancer status: the importance of risk of recurrence was higher in younger participants but was not influenced by cancer group. We also observed higher aversion to a stoma in younger compared with older participants. In contrast, the NC group were less averse to a permanent stoma than the CNS group. This suggests that differences in age between the NC and cancer groups did not unduly influence our findings. The NC group also reported lower levels of self‐reported health than the two cancer groups, and this may have influenced their perceived ability to cope with bowel dysfunction or a permanent stoma. However, the two cancer groups reported similar levels of self‐rated health, but a different pattern of preferences was observed when comparing the NC with the CS group and the NC with the CNS group. Again, this suggests the preferences of the NC group cannot be solely attributed to differences in self‐rated health. Finally, while we excluded patients with metastatic disease, we lacked data on cancer recurrence following treatment. As a result, we could not assess the impact of actual recurrence on treatment preferences, notably in relation to risk of recurrence.

## CLINICAL IMPLICATIONS AND CONCLUSION

Experience of a stoma or LARS resulted in higher tolerance of those treatment outcomes. People are often highly fearful of a stoma, and those undergoing sphincter‐preserving surgery are often more focused on the imminent surgery and being cancer‐free rather than bowel dysfunction [13]. Information from patients who have experienced different outcomes following rectal cancer surgery would help newly diagnosed patients be more prepared for the effects of treatment and assist informed decision‐making where patients have a choice.

## AUTHOR CONTRIBUTIONS

**Anne Miles:** Conceptualization; data curation; formal analysis; visualization; methodology; investigation; supervision; project administration; resources; funding acquisition; validation; software; writing – original draft; writing – review and editing. **Robert James Campbell Steele:** Conceptualization; funding acquisition; methodology; supervision; writing – original draft; writing – review and editing. **Gemma Hutton:** Investigation; project administration; resources; writing – original draft; writing – review and editing. **Stephen Morris:** Conceptualization; data curation; formal analysis; funding acquisition; methodology; resources; software; supervision; validation; visualization; writing – original draft; writing – review and editing.

## FUNDING INFORMATION

This project was funded by Bowel Research UK.

## CONFLICT OF INTEREST STATEMENT

The authors declare no conflicts of interest.

## ETHICS STATEMENT

Ethical approval and patient consent were both obtained.

## Supporting information


**Data S1.** Supporting Information.

## Data Availability

The data that support the findings of this study are available from the corresponding author upon reasonable request.
