# Supplementary material for: Preferences for treatment outcomes in rectal cancer: A discrete choice experiment among patients and healthy volunteers
Source: Colorectal Dis. 2025 Feb 9;27(2):e70021. doi: 10.1111/codi.70021 (PMC11808228; doi:10.1111/codi.70021)
Supplement: Supplementary file 1 — Data S1. Supporting Information. [file CODI-27-0-s001.docx]

**Supplementary tables**

**Table S1: Results of conditional logit regression analysis by group, with bowel attributes converted into LARS scores (coefficients and 95% confidence intervals).**

| **Attributes and attribute levels** | All participants | Cancer no stoma | Cancer with stoma | No cancer | Older | Younger |
| --- | --- | --- | --- | --- | --- | --- |
| LARS scores categorised |  |  |  |  |  |  |
| [No LARS] | - | - | - | - | - | - |
| Minor LARS | -0.296 ^a^  (-0.384, -0.208) | -0.237 ^a^  (-0.392, -0.082) | -0.338 ^a^  (-0.486, -0.189) | -0.505 ^a,1^  (-0.702, -0.308) | -0.197 ^a^  (-0.313, -0.081) | -0.437 ^a,3^  (-0.580, -0.295) |
| Major LARS | -0.337 ^a^  (-0.420, -0.254) | -0.157 ^a^  (-0.297, -0.017) | -0.496 ^a,1^  (-0.647, -0.345) | -0.557 ^a,1^  (-0.737, -0.378) | -0.243 ^a^  (-0.355, -0.131) | -0.470 ^a,3^  (-0.598, -0.342) |
| Risk of recurrence | -7.367 ^a^  (-8.027, -6.706) | -7.599 ^a^  (-8.734, -6.464) | -8.335 ^a^  (-9.591, -7.079) | -7.106 ^a^  (-8.557, -5.656) | -6.647 ^a^  (-7.545, -5.750 | -8.464 ^a,3^  (-9.548, -7.380) |
| Presence of stoma |  |  |  |  |  |  |
| [No] | - | - | - | - | - | - |
| Yes | -0.741 ^a^  (-0.794, -0.688) | -1.281 ^a^  (-1.374, -1.188) | -0.003^1^  (-0.094, 0.087) | -0.906 ^a,1, 2^  (-1.020, -0.792) | -0.594 ^a^  (-0.663, -0.524) | -0.944 ^a,3^  (-1.028, -0.860) |
| Observations/ respondents | 13,968 / 873 | 5,952/ 372 | 4,304/ 269 | 3264 / 204 | 7,568 | 6,400 |
| Likelihood ratio chi-square  (p value) | Wald chi-square 1115.13 (<0.0001) | 1105.10 (p<0.0001) | 284.48 (p<0.0001) | 422.85 (p<0.0001) | 572.81  (<0.0001) | 889.61  (<0.0001) |

^a^ Significantly different from reference category at p<0.05

^1^ Significantly different from the equivalent level in the CNS group

^2^ Significant difference between CS and NC groups

^3^ Significantly different from the equivalent level in older patients.

**Table S2: Results of conditional logit regression analysis by age, for all attributes (coefficients and 95% confidence intervals) and marginal rates of substitution.**

| **Attributes and attribute levels** | **Older**  >65 years | **Younger**  <=65 |
| --- | --- | --- |
| Leakage of wind |  |  |
| [No, never] | - | - |
| Yes, less than once per week | -0.107 ^a^  (-0.199, -0.015) | -0.060  (-0.172, 0.053) |
| Yes, at least once per week | -0.165 ^a^  (-0.271, -0.058) | -0.109  (-0.233, 0.015) |
|  |  |  |
| Leakage of liquid stool |  |  |
| [No, never] | - | - |
| Yes, less than once per week | -0.235 ^a^  (-0.328, -0.143) | -0.465 ^a, 1^  (-0.574, -0.356) |
| Yes, at least once per week | -0.304 ^a^  (-0.413, -0.195) | -0.621 ^a,b,2^  (-0.761, -0.481) |
|  |  |  |
| Need to empty bowels again within one hour of emptying (incomplete emptying) |  |  |
| [No, never] | - | - |
| Yes, less than once per week | -0.073  (-0.164, 0.019) | -0.150 ^a^  (-0.261, -0.038) |
| Yes, at least once per week | 0.000  (-0.110, 0.111) | -0.191 ^a, 1^  (-0.323, -0.059) |
|  |  |  |
| Need to rush to the toilet |  |  |
| [No, never] | - | - |
| Yes, less than once per week | -0.169 ^a^  (-0.260, -0.077) | -0.307 ^a^  (-0.414, -0.199) |
| Yes, at least once per week | -0.144 ^a^  (-0.252, -0.036) | -0.320 ^a, 1^  (-0.455, -0.186) |
|  |  |  |
| Number of times open bowel |  |  |
| [1 to 3 times per day] | - | - |
| Less than once per day | -0.012  (-0.136, 0.112) | -0.135  (-0.296, 0.027) |
| 4 to 7 times per day | -0.360 ^a^  (-0.483, -0.238) | -0.329 ^a^  (-0.477, -0.181) |
| More than 7 times per day | -0.523 ^a,b^  (-0.666, -0.380) | -0.668 ^a,b^  (-0.842, -0.494) |
|  |  |  |
| Risk of recurrence | -6.550 ^a^  (-7.406, -5.692) | -8.729 ^a, 1^  (-9.814, -7.644) |
| Presence of a stoma |  |  |
| [No] | - | - |
| Yes | -0.632 ^a^  (-0.704, -0.559) | -1.000 ^a, 1^  (-1.087, -0.908) |
| Observations/ respondents | 7,568 | 6,400 |
| **Marginal rates of substitution** |  |  |
| Increase in risk of recurrence to avoid a stoma | 9.6% | 11.4% |
| Increase in LARS score to avoid a stoma | 44.1 | 37.8 |
| Increase in risk of recurrence to avoid minor LARS | 2.9% | 5.2% |
| Increase in risk of recurrence to avoid major LARS | 3.7% | 5.6% |

^a^ Significantly different from reference category

^b^ Significantly different from the level “yes, less than once a week”/ “4 to 7 times per day”

^1^ Significantly different from the equivalent level in older patients

**Table S3: Overview of how bowel function severity predicted treatment preference across groups**

|  | **All participants** | **Cancer no stoma**  **(CNS)** | **Cancer stoma**  **(CS)** | **No cancer**  **(NC)** | **Older** | **Younger** |
| --- | --- | --- | --- | --- | --- | --- |
| **Severity level significant** | Number of times open bowels  Accidental leakage of liquid stool | Number of times open bowels | Number of times open bowels | Number of times open bowels  Accidental leakage of liquid stool | Number of times open bowels | Number of times open bowels  Accidental leakage of liquid stool |
| **Presence but not severity level significant** | Need to rush to the toilet  Accidental leakage of wind  Need to empty bowels again within one hour of opening  LARS minor/major | Accidental leakage of liquid stool  Need to rush to the toilet  LARS minor/major | Accidental leakage of liquid stool  Need to rush to the toilet  Accidental leakage of wind  LARS minor/major | Need to empty bowels again within one hour of opening  Need to rush to the toilet  LARS minor/major | Accidental leakage of liquid stool  Need to rush to the toilet  Accidental leakage of wind  LARS minor/major | Need to empty bowels again within one hour of opening  Need to rush to the toilet  LARS minor/major |
| **Not a significant predictor of preference** | - | Accidental leakage of wind  Need to empty bowels again within one hour of opening | Need to empty bowels again within one hour of opening | Accidental leakage of wind | Need to empty bowels again within one hour of opening | Accidental leakage of wind |

**Table S4: All participants, bowel variables converted into total LARS scores**

| **Attributes and attribute levels** | **All participants** | **Cancer no stoma**  **(CNS)** | **Cancer with stoma**  **(CS)** | **No cancer**  **(NC)** | **Older** | **Younger** |
| --- | --- | --- | --- | --- | --- | --- |
| **LARS continuous** | -0.01826 ^a^  (-0.02166 to -0.01486) | -0.01019 ^a^  (-0.01593 to -0.00445) | -0.02525 ^a, 1^  (-0.03141 to -0.0191) | -0.0278 ^a, 1^  (-0.03519 to -0.0204) | -.0135165 ^a^  (-.0180566 to  -.0089765) | -.0251488 ^a, 3^  (-.0304977 to  -.0197999) |
|  |  |  |  |  |  |  |
| **Risk of recurrence** | -7.34841 ^a^  (-7.98388 to -6.71294) | -7.70334 ^a^  (-8.80517 to -6.60152) | -8.17175 ^a^  (-9.35434 to -6.98916) | -7.15817 ^a^  (-8.50129 to -5.81504) | -6.611395 ^a^  (-7.468158 to  -5.754632) | -8.483741 ^a, 3^  (-9.52488 to  -7.442602) |
| **Presence of stoma** |  |  |  |  |  |  |
| **[No]** | - |  |  |  |  |  |
| **Yes** | -0.74534 ^a^  (-0.7987 to -0.69199) | -1.28075 ^a^  (-1.37369 to -1.18782) | -0.00953 ^1^  (-0.09996 to 0.080906) | -0.9127 ^a, 1, 2^  (-1.02821 to -0.79719) | -.596697 ^a^  (-.6662977 to  -.5270963) | -.9512963 ^a, 3^  (-1.035858 to  -.866735) |
| **Observations/ respondents** | 13,968 | 5,952 | 4,304 | 3,264 | 7,568 / | 6,400 / |
| **Likelihood ratio chi-square**  **(p value)** | Wald chi-square 1111.46  (<0.0001) | 1104.01  (<0.0001) | 299.36  (<0.0001) | 419.52  (<0.0001) | 581.35 (<0.0001) | 894.47  (<0.0001) |

^a^ Significant predictor

^1^ Significantly different from CNS group

^2^ Significant difference between CS and NC groups

^3^ Significantly different from the equivalent level in older patients.

**Table S5: Rank of importance of treatment attributes across different groups**

| **Rank order of importance** | **All participants** | **Cancer no stoma**  **(CNS)** | **Cancer with stoma (CS)** | **No cancer (NC)** | **Older** | **Younger** |
| --- | --- | --- | --- | --- | --- | --- |
| 1 | ***Risk of recurrence*** | ***Stoma*** | ***Risk of recurrence*** | ***Risk of recurrence*** | ***Risk of recurrence*** | ***Risk of recurrence ^b^*** |
| 2 | ***Stoma*** | ***Risk of recurrence*** | ***Number of times open bowels ^1^*** | ***Stoma ^1,2^*** | ***Stoma*** | ***Stoma ^b^*** |
| 3 | ***Number of times open bowels*** | ***Number of times open bowels*** | ***Accidental leakage of liquid stool*** | ***Number of times open bowels*** | ***Number of times open bowels*** | ***Number of times open bowels*** |
| 4 | ***Accidental leakage of liquid stool*** | ***Accidental leakage of liquid stool*** | ***Need to rush to the toilet ^1^*** | ***Accidental leakage of liquid stool ^1^*** | ***Accidental leakage of liquid stool*** | ***Accidental leakage of liquid stool ^b^*** |
| 5 | ***Need to rush to the toilet*** | Need to rush to the toilet | ***Accidental leakage of wind ^1^*** | ***Need to rush to the toilet ^1^*** | ***Need to rush to the toilet*** | ***Need to rush to the toilet ^b^*** |
| 6 | ***Accidental leakage of wind*** | Need to empty bowels again within one hour of opening | Need to empty bowels again within one hour of opening | ***Need to empty bowels again within one hour of opening ^1^*** | ***Accidental leakage of wind*** | ***Need to empty bowels again within one hour of opening ^b^*** |
| 7 | ***Need to empty bowels again within one hour of opening*** | Accidental leakage of wind | Stoma ^1^ | Accidental leakage of wind | Need to empty bowels again within one hour of opening | Accidental leakage of wind |

In bold and italics = significant predictors of preference within that group of participants.

^1^ Significant difference from CNS

^2^ Significant difference between CS and NC groups

^b^ Significant difference between older and younger groups

**Figure S1: Predicted probability of choosing no stoma with varying levels of LARS and risk of recurrence, compared to choosing a stoma with a 1% risk of recurrence - Older participants**

**Figure S2: Predicted probability of choosing no stoma with varying levels of LARS and risk of recurrence, compared to choosing a stoma with a 1% risk of recurrence - Younger participants**
